# Treatment of Leukemic Blood Samples with Granulocyte-Macrophage-Colony-Stimulating-Factor Combined with Prostaglandin E1 Is Associated with Reduced Frequencies of Tolerogenic Dendritic Cells and Increased Cytotoxicity Against Autologous Blasts

**DOI:** 10.3390/biomedicines14061279

**Published:** 2026-06-04

**Authors:** Anne Hartz, Lin Li, Hazal Aslan Rejeski, Elena Pepeldjiyska, Elias Rackl, Tobias Baudrexler, Peter Bojko, Jörg Schmohl, Andreas Rank, Christoph Schmid, Helga Schmetzer

**Affiliations:** 1 Working-Group: Immune-Modulation, Department for Hematopoietic Cell Transplantation, Medical Department 3, Klinikum Grosshadern, Ludwig-Maximilians-University, 81377 Munich, Germany; 2 Bavarian Cancer Research Center (BZKF), 91054 Erlangen, Germany; 3 Department of Hematology and Oncology, Rotkreuzklinikum Munich, 80634 Munich, Germany; 4 Department of Hematology and Oncology, Diakonieklinikum Stuttgart, 70176 Stuttgart, Germany; 5 Department of Hematology and Oncology, University Hospital of Augsburg, 86156 Augsburg, Germany

**Keywords:** PGE1, GM-CSF, AML, blast modulation, immune therapy, dendritic cells, tolerogenic dendritic cells

## Abstract

**Background:** Acute myeloid leukemia (AML) is characterized by reduced antileukemic effector cells and increased immunosuppressive cell populations. Leukemia-derived dendritic cells (DC_leu_), generated from 18 leukemic whole blood (WB) ex vivo using ‘Kit-M’ (clinically approved: GM-CSF + PGE1), lead to improved cytotoxicity against autologous blasts after mixed lymphocyte culture (MLC) with patients’ T-cells. **Methods:** We studied Kit-M-mediated effects on frequencies of tolerogenic, immunosuppressive DC (DC_tol_) and correlated findings with ex vivo-achieved antileukemic effects (increased intracellular IFNγ production/degranulation, blast lysis) and patients’ clinical characteristics. **Results:** We show significantly decreased frequencies of DC_tol_ (and increased frequencies of mature DC_leu_) without induced blast proliferation in Kit-M treated vs. untreated WB samples. After T-cell-enriched MLC with Kit-M pretreated vs. not pretreated, WB frequencies of regulatory (CD152+ T-cells) were significantly decreased, while ‘activated’ (IFNγ+, degranulating) non-naive, proliferating, memory, CD154+) T-cells, as well as NK and CIK-cells were (significantly) increased. We found a (significant) positive correlation of achieved improved blast lysis, frequencies of DC_leu_ and ‘activated’ (IFNγ+/degranulating) T- or NK/CIK cells, and a (significant) negative correlation with frequencies of DC_tol_ and regulatory (CD152+ T-cells). Kit-M treatment of leukemic WB increases DC_leu_ and decreases DC_tol_, correlating with improved immune reactions/improved cytotoxicity against autologous blasts, and downregulated suppressive T-cells in samples before or after MLC. **Conclusions:** These findings demonstrate the potential of Kit-M (using clinically approved drug compositions) to treat AML patients to potentially overcome the immunosuppressive tumor microenvironment, leading to improved antileukemic responses—thereby stabilizing remission of the disease in AML patients.

## 1. Introduction

Acute myeloid leukemia (AML), the most common adult acute leukemia, is a clonal malignancy leading to infiltration of the bone marrow, blood, and other tissues by proliferative, abnormally differentiated cells of the hematopoietic system [1,2]. General therapeutic strategies in patients suffering from AML have not changed significantly within the last few decades, consisting of chemotherapy with or without allogeneic hematopoietic-cell transplantation [3]. The 5-year survival rates in older patients (age 60 years and older), representing the main cohort of AML patients who receive intensive chemotherapy, are still poor (<10–15% of patients) [4]. Recently, new immuno- and chemotherapeutic therapies are being tested for their capability to eradicate leukemic cells and/or to sustain remission: cellular therapies (specific chimeric antigen receptor (CAR)-T-cells), antibodies (BiTEs, checkpoint antibodies), tyrosine kinase inhibitors or antigen-presenting cells (APCs) such as dendritic cells (DCs), the most potent APCs [4,5,6,7].

DCs, representing a heterogeneous group of cells that are different in origin, function, phenotype and localization, serve as a crucial link between the innate and adaptive immunity [8,9]. Besides their immunogenic properties, such as efficient immune cell priming and stimulating immune cells, DCs can also acquire tolerogenic properties depending on different stimuli (e.g., interleukin (IL)-10, transforming growth factor (TGF)- β) they are exposed to [9,10,11,12]. On the one hand, the lack of immunological tolerance can cause the development of inflammatory and autoimmune diseases [13]. On the other hand, DCs were found to exert a tolerogenic phenotype, characterized by decreased antigen cross-presentation and induced regulatory T-cell (T_reg_) differentiation, within the context of cancer [14,15]. This immunosuppressive function of tolerogenic dendritic cells (DC_tol_) is mediated through metabolic and signaling pathways, including the immunoregulatory activity of the indoleamine-pyrrole-2,3-deoxygenase (IDO) enzyme and inducible nitric oxide synthase (iNOS) activity. Furthermore, DC_tol_ release inhibitory cytokines such as IL-10 and TGF-β and express various inhibitory receptors, including programmed death-ligand 1 (PD-L1), PD-L2, programmed death 1 (PD-1), inhibitory Ig-like transcripts (e.g., ILT3), and cytotoxic T-lymphocyte-associated protein 4 (CTLA-4), further contributing to the suppression of anti-tumor immunity [9,16,17,18]. ILT3 serves as a canonical inhibitory receptor associated with tolerogenic differentiation [19,20]. PD-1 and CTLA4, although classically attributed to T cells, have also been reported to be expressed on DCs under immunoregulatory conditions and are associated with reduced activation capacity and the establishment of inhibitory signaling programs [17,21,22]. Interleukin-3 receptor (IL3RA) enables the identification of dendritic cell subsets, such as plasmacytoid DC, with known tolerogenic potential [23,24].

DC-based immunotherapy represents a biologically promising strategy in leukemia, given the central role of DCs in antigen presentation and the induction of leukemia-specific adaptive and innate (effector and memory) responses [25]. Current approaches primarily include ex vivo-generated monocyte-derived DC vaccines loaded with leukemia-associated antigens (LAAs) and leukemia-derived DCs (DC_leu_), which present the full antigenic repertoire of leukemic blasts [26,27]. Early-phase clinical trials have demonstrated favorable safety profiles and the induction of antigen-specific immune responses, although clinical efficacy remains limited in patients with high-disease burden [28,29].

Major challenges in AML treatment include the intrinsically low immunogenicity of leukemic cells due to their low tumor mutational burden, antigen heterogeneity and immune escape, and the establishment of an immunosuppressive microenvironment characterized by regulatory T-cells, inhibitory cytokines, and immune checkpoint activation [30,31]. To address these limitations, strategies that enhance DC-mediated functionality are under investigation. In this context, the combination of granulocyte–macrophage colony-stimulating factor (GM-CSF) in combination with prostaglandin E1 (PGE1), ‘Kit-M’, has emerged as a promising approach, as it was already shown to enable the efficient conversion of leukemic blasts into functionally competent DC_leu_, improves DC yield and maturation, enhances cytotoxic cell activation, and reduces regulatory T-cell–mediated immunosuppression [18,32,33,34,35]. Nevertheless, despite these advances, a deeper mechanistic understanding of the ‘mode of action’ of DC-mediated reactions, e.g., of DC subset differentiation—particularly the balance between immunogenic and tolerogenic DCs—and their interaction as well as the clinical impact of different DC/DC_leu_ mediated strategies is still lacking [36].

Our research aims to contribute to a better understanding of DC biology and to overcome immune escape. We hypothesized that targeting immune escape mechanisms—especially the reduction in tolerogenic/suppressive immune effector cells—may enhance antileukemic immune responses. Therefore, we analyzed especially frequencies of (tolerogenic) DC subsets in Kit-M treated vs. untreated whole blood (WB) from AML patients (and healthy donors). Moreover, we quantified frequencies of (leukemia-specific) innate and adaptive immune cells before or after mixed lymphocyte culture (MLC) in Kit-M-treated vs. untreated samples. Finally, we correlated results after DC and MLC with a special focus on tolerogenic DC (DC_tol_) and their role in achieving antileukemic functionality and patients’ clinical data (European LeukemiaNet (ELN) risk groups and response to induction therapy).

## 2. Materials and Methods

### 2.1. Sample Collection

Patient samples were collected between 11 September 2019 and 4 December 2020. All patients were included before chemotherapeutic treatment.

Sample collection was conducted after receiving written informed consent of blood donors and in accordance with the World Medical Association Declaration of Helsinki and the local ethics committee of the Ludwig-Maximilian-University-Hospital Munich (Pettenkoferstrasse 8a, 80336 Munich, Germany (Vote 19-034, 11 September 2019, date of ethical approval in Institutional Review Board Statement). Heparinized peripheral WB samples were taken from patients in acute (blast-containing) phases of AML (*n* = 18) and from healthy donors (*n* = 5). Samples were provided by the University Hospitals of Augsburg, Stuttgart and Munich.

### 2.2. Patients’ Characteristics

AML patients were characterized and classified in accordance with the FAB classification (M1–M7), the etiology (primary, secondary), the stage of the disease, the blast phenotype and blast frequencies in peripheral blood [29,37]. 12 patients were diagnosed with primary (p) AML, 6 with secondary (s) AML after prior myeloproliferative neoplasia (MPN) or myelodysplastic syndrome (MDS). Patients presented at first diagnosis (*n* = 16), at relapse (*n* = 1) or at relapse after stem cell therapy (*n* = 1). Patients with AML at initial diagnosis were stratified into low (*n* = 9), intermediate (*n* = 4), and high (*n* = 5) risk categories in accordance with ELN criteria [3]. Treatment response was assessed based on achieving or not achieving complete remission (defined as <5% blasts in the bone marrow and absence of circulating blasts in peripheral blood 30 days after initiation of induction therapy). The mean age of AML patients was 62.6 (range 22–82) years; the age of healthy volunteers was 26 (range 24–28) years. The female: male ratio of patients was 1:0.6 in AML patients and 1:0.7 in healthy donors. Further details are given in Supplementary Table S1.

The cellular composition of the patients’ blood samples was on average: 42.01 (range 9–85)% blasts (BLA) in peripheral blood, 2.70 (range 0.32–6.89)% B-cells, 10.56 (range 1.75–27.10)% T-cells, 2.38 (range 0.20–7.41)% natural killer (NK)-cells, 1.74 (range 0.32–3.85)% cytokine-induced killer (CIK)-cells and 4.91 (range 0.80–14.40)% monocytes. In case of aberrant antigen expression, frequencies of NK-, CIK-, T-, B-cells or monocytes were not calculated. Abbreviations of all cell types are given in Supplementary Table S2.

### 2.3. Flow Cytometry and Sample Preparation

Frequencies of cells were quantified by flow cytometry [38]. Flow cytometry was conducted using a panel of mouse monoclonal antibodies (mAbs) directly conjugated to fluorescein-isothiocyanate (FITC), phycoerythrin (PE), phycoerythrin/cyanine 7 (PE/Cy 7) and allophycocyanin (APC) to analyze cell frequencies before and after cultures. The antibodies used for flow cytometric staining are listed in the Supplementary Materials.

Flow cytometric analyses were performed by suspending samples in phosphate-buffered saline (PBS) supplemented with 5% fetal bovine serum (FBS, Bio&Sell, Feucht, Germany). Cell suspensions were incubated with mAbs for 15 min, followed by washing, centrifugation, and resuspension in 100 µL PBS. WB samples were additionally treated with a lysing buffer (BD Biosciences, San Jose, CA, USA) in accordance with the manufacturer’s protocol.

Flow cytometric analyses were conducted using a four-color FACSCalibur flow cytometer (BD Biosciences, 2350 Qume Drive, San Jose, CA, USA) in combination with CellQuest Pro 6.1 software (Becton Dickinson, Heidelberg, Germany). Isotype controls were routinely acquired in parallel with the corresponding antibody stainings and used to define background fluorescence and positivity thresholds. Identical forward scatter/side scatter and fluorescence gating parameters were consistently applied across isotype control samples and specifically stained samples. Isotype controls were experimentally measured and not inferred solely from the applied gating strategy.

Abbreviations for all analyzed cell populations are summarized in Supplementary Table S2. Further details are given in the Supplementary Materials.

All cell culture experiments, including DC cultures, MLCs, Intracellular Cytokine Assay (ICA), Degranulation Assay (Deg) and a cytotoxicity fluorolysis assay (CTX), were conducted under standard laboratory conditions (37 °C, 21% O_2_ and 5% CO_2_).

### 2.4. Dendritic Cell Culture

Generating DC and DC_leu_ from leukemic and healthy WB was performed using the established DC/DC_leu_-generating protocol with a specific immune modulatory Kit (Kit-M: 800 U/mL GM-CSF + 1 µg/mL PGE1; Kit-M-treated DC culture (DC(M)), as described [39,40,41]. Cultures without immunomodulators served as negative controls (DC(C)). Cells were harvested after 7–9 days of culture. Further descriptions of the technical procedures are given in the Supplementary Materials. The analyzed cell subtypes are summarized in Supplementary Table S2.

Flow cytometric evaluation of leukemic blasts, DC, DC_leu_ and mature DCs (DC_mat_) was performed using an established and refined gating strategy as described [40,42,43]. DC_leu_ were identified based on the simultaneous expression of at least one leukemic blast marker, including lineage-aberrant markers (e.g., CD117), together with at least one dendritic cell marker that is not present on naïve blasts (e.g., CD80), Supplementary Figure S4. The maturation status of DCs and DC_leu_ was determined by CCR7 co-expression.

### 2.5. Mixed Lymphocyte Culture

T-cell enriched MLCs, containing various immunoreactive cells, were stimulated with a stimulator cell suspension pretreated with or without Kit-M [32]. Initially, 1 × 10^6^ thawed patient-derived CD3^+^ T-cells were co-cultured with interleukin 2 (IL-2) and a stimulator cell suspension containing approximately 2.5 × 10^5^ generated DC/DC_leu_, generated with vs. without Kit-M as control. After 6–7 days of culture, cells were harvested, and their subtypes were quantified and used for subsequent experiments such as Deg, ICA and CTX.

### 2.6. Functional Analyses: Intracellular Cytokine Assay, Degranulation Assay and Cytotoxicity Fluorolysis Assay

Functional cell responses were assessed using ICA and Deg assays, quantifying IFNγ producing or degranulating, potentially leukemia-specific immune cell subsets, as described [32,39]. Blast lytic activity of T cell–enriched immunoreactive cells was assessed after MLC with Kit-M-treated WB cultures. For this purpose, a defined fraction of MLC containing 1 × 10^6^ T cells (as effector cells) was co-cultured with 1 × 10^6^ thawed autologous leukemic blasts (as target cells). As a control, effector and target cells were cultured separately under identical conditions and combined only immediately prior to flow cytometric analysis. The lytic activity against blasts (‘blast lysis’) was determined by calculating the percentage difference in viable 7AAD–negative blast target cells between co-cultured and separately cultured effector and target cells, as previously described [32,39]. For a more detailed assessment, ‘improved lysis’ was calculated as the percentage difference between the achieved lysis of Kit-M pretreated WB vs. untreated WB in every given case, as described before [32,35]. Details of the technical procedures are provided in the Supplementary Materials.

### 2.7. Statistical Methods

Data are presented as mean ± standard deviation (SD). All comparisons between Kit-M–treated and untreated control conditions were performed using two-tailed paired Student’s *t*-tests, as samples originated from the same patients and therefore represented dependent observations. Because multiple cell subtypes were analyzed in parallel, *p*-values were adjusted for multiple comparisons using the Benjamini–Hochberg false discovery rate (FDR) procedure. Adjusted *p*-values < 0.05 were considered statistically significant.

Correlations were assessed using the Pearson correlation coefficient. Correlation was defined as ‘low’ with r values 0.30 to 0.50 (−0.30 to −0.50), as ‘moderate’ with r values 0.50 to 0.70 (−0.50 to −0.70) and as ‘high correlation’ with r values 0.70 to 1.00 (−0.70 to −1.00). For selected analyses and correlations, differences in cell subset frequencies are expressed as relative changes (Δ) between Kit-M–treated and untreated conditions.

Additional post hoc power analyses were performed for the principal comparisons underlying the main findings. While the available sample size was considered adequate for the main comparisons, subgroup analyses with smaller sample sizes were considered exploratory and interpreted with caution.

Statistical analyses and figures were applied with GraphPad Prism 9 (GraphPad Software, San Diego, CA, USA) and Excel 2010 (Microsoft, Redmond, WA, USA).

## 3. Results

We quantified immune cells, focusing on tolerogenic dendritic cells (DC_tol_), after DC and mixed lymphocyte culture ex vivo. We then correlated immune cell profiles with (ex vivo) achieved blastolytic functionality and patients’ clinical data (details of patients’ characteristics are provided in Section 2 and in Supplementary Materials).

### 3.1. Composition of DCs (e.g., DC, DC_leu,_ DC_mat_) After Kit-M Treatment Healthy and Leukemic WB

#### 3.1.1. Significantly Higher Frequencies of Mature DCs and DC_leu_ Generated with Kit-M from Leukemic WB Without Induction of Blasts

WB samples, from AML patients and healthy donors, were cultured for 7 days with or without Kit-M as control. After 7 days of DC culture, flow cytometric analyses were conducted using flowcytometry, as described in Section 2.3 and Section 2.4. Results with Kit-M (DC(M)) and without added Kit as control (DC(C)) are given.

**Figure 1:**
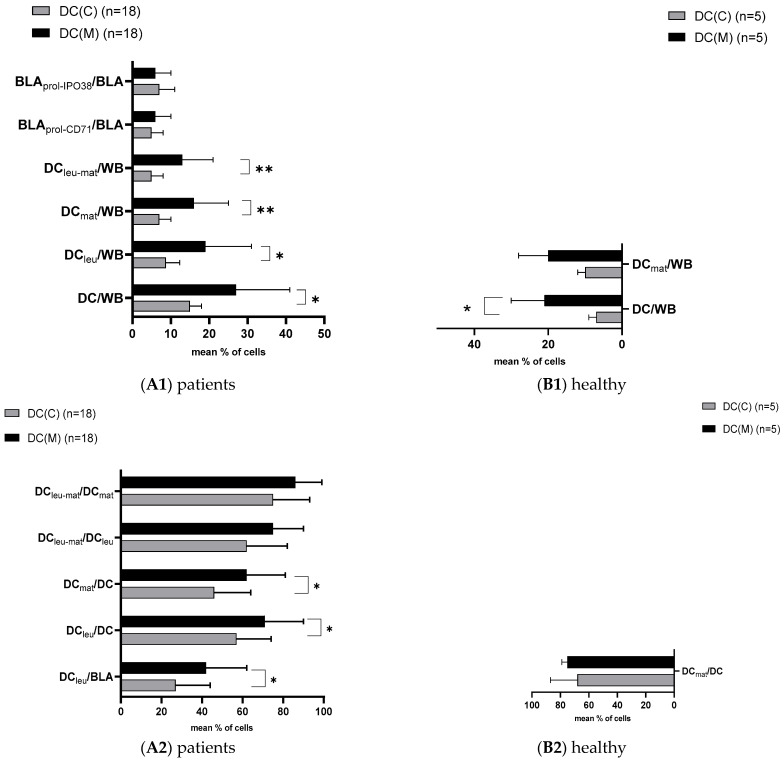
Effects of Kit-M on the generation of (mature) DC/DC_leu_ in leukemic and healthy WB. Leukemia-derived dendritic cells (DC_leu_) were generated using leukemic (**A1**,**A2**) and healthy (**B1**,**B2**) whole blood (WB) with (DC(M)) and without (DC(C)) Kit-M treatment; WB samples were cultivated with and without (control) Kit-M for 7 days. Mean frequencies ± standard deviation of (mature) DC(_leu_) in (**A**) AML and (**B**) healthy samples within all cells (**A1**,**B1**) and other DC(_leu_) subpopulations (**A2**,**B2**), quantified by flow cytometry, are given. Paired *t*-test was applied: Results were regarded as significantly different (*) with a *p*-value < 0.05 and as highly significant (**) with a *p*-value < 0.005, applying a paired *t*-test. Abbreviations: DC_leu_: leukemia-derived DC; DC_mat_: mature DC; DC_leu-mat_: mature DC_leu_; BLA: blasts; BLA_prol-CD71/IPO38_: proliferating blasts; *n*: number of cases; cell subtypes are given in Supplementary Table S2.

We generated significantly higher frequencies of mature DCs (DC_mat_) in Kit-M-treated leukemic samples compared to control (%DC_mat_/WB: Kit-M: 13.97 ± 7.76 vs. control 6.69 ± 3.52, *p* < 0.005; %DC_mat_/DC: Kit-M: 62.47 ± 18.57 vs. control 46.12 ± 17.98, *p* < 0.05). In DC(M), we also found significantly higher frequencies of DC_leu_ compared to control (%DC_leu_/WB: Kit-M: 19.37 ± 12.05 vs. control 8.41 ± 2.96, *p* < 0.05; %DC_leu_/DC: Kit-M: 71.18 ± 18.60 vs. control 58.33 ± 19.27, *p* < 0.05) (Figure 1(A1,A2)). No induced proliferation of blast cells was observed under the influence of Kit-M compared to control (Figure 1(A1), upper part). Abbreviations of cell subtypes are given in Supplementary Table S2.

Similar results were also found in healthy blood (*n* = 5): (Significantly) Higher frequencies of (mature) DC were observed under the influence of Kit-M compared to control, without inducing proliferation of monocytes (%DC/WB: Kit-M: 21.16 ± 8.82 vs. control 6.62 ± 2.41, *p* < 0.05) (Figure 1(B1,B2) right side).

#### 3.1.2. Lower Frequencies of DC_tol_ in Leukemic Kit-M Treated vs. Untreated WB

We quantified DC_tol_ after culture using and combining various markers: ILT3, CTLA4, PD1 and IL3RA. of DC subsets, such as plasmacytoid DC, with known tolerogenic potential [23,24].

**Figure 2:**
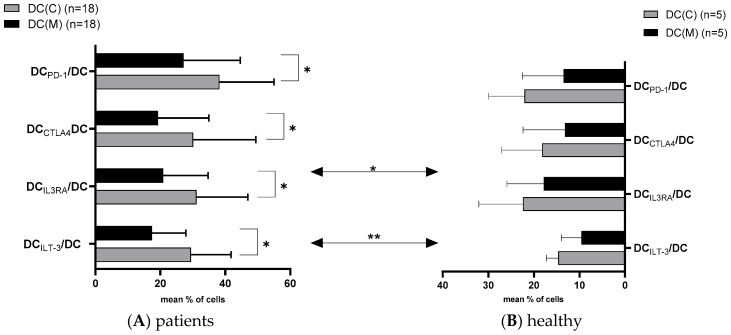
Effects of Kit-M on frequencies of DC_tol_ in leukemic and healthy WB. WB samples were cultivated with and without (control) Kit-M for 7 days. Mean frequencies ± standard deviation in Kit-M treated (DC(M)) vs. untreated (DC(C)) (**A**) AML and (**B**) healthy samples of DC_tol_ (DC_ILT-3_, DC_IL3RA_, DC_CTLA4_, DC_PD-1_) within DC, quantified by flow cytometry, are given. Paired *t*-test was applied: Results were regarded as significantly different (*) with a *p*-value < 0.05 and as highly significant (**) with a *p*-value < 0.005. Abbreviations: DC_ILT-3_: ILT-3 expressing DC; DC_IL3RA_: IL3RA expressing DC; DC_CTLA4_: CTLA4 expressing DC; DC_PD-1_: PD-1 expressing DC; *n*: number of cases. Double-sided arrows give (significant) differences between defined cell subtypes in patients vs. healthy WB samples. Abbreviations for subtypes are given in Supplementary Table S2.

We found highly significantly lower frequencies of ILT-3 expressing DC_tol_ in Kit-M treated leukemic WB compared to control (%DC_ILT-3_/DC: Kit-M: 17.41 ± 10.46 vs. control: 29.39 ± 12.39, *p* < 0.05). In addition, we also found significantly lower frequencies of IL3RA–(%DC_IL3RA_/DC: Kit-M: 20.86 ± 13.83 vs. control 31.11 ± 15.84, *p* < 0.05), CTLA4–(%DC_CTLA4_/DC: Kit-M: 19.24 ± 15.68 vs. control 30.12 ± 19.28, *p* < 0.05) and PD-1-expressing DC_tol_ (%DC_PD-1_/DC: Kit-M: 27.14 ± 17.42 vs. control 38.16 ± 16.81, *p* < 0.05) in Kit-M treated leukemic WB compared to control (Figure 2A).

In healthy Kit-M-treated WB, frequencies of DC_tol_ subtypes were reduced (Figure 2B). Comparing frequencies of DC_tol_ in AML vs. healthy WB samples with vs. without treatment with Kit-M, we found (significantly) higher frequencies in AML compared to healthy samples (Figure 2A vs. Figure 2B).

### 3.2. Comparison of (Leukemia-Specific) Immune Cells After Stimulation in T-Cell Enriched Mixed Lymphocyte Culture Using Kit-M Treated vs. Untreated WB

To further evaluate the DC/DC_leu_-mediated stimulation of immunoreactive cells, we compared cell subtype distributions within cell fractions before (MLC(UC)) and after T-cell-enriched MLC using Kit-M treated (MLC(M)) as stimulator cells vs. untreated WB (MLC(CC)). Therefore, an aliquot of (DC/DC_leu_ containing) cells after culture with vs. without Kit-M, was cocultured with thawed autologous patients’ T-cells, stimulated with IL-2 and cultured for 6–7 days.

#### 3.2.1. (Significant) Production of (Leukemia-Specific) Immunoreactive Cells in Kit-M Pretreated vs. Untreated WB After MLC

**Figure 3:**
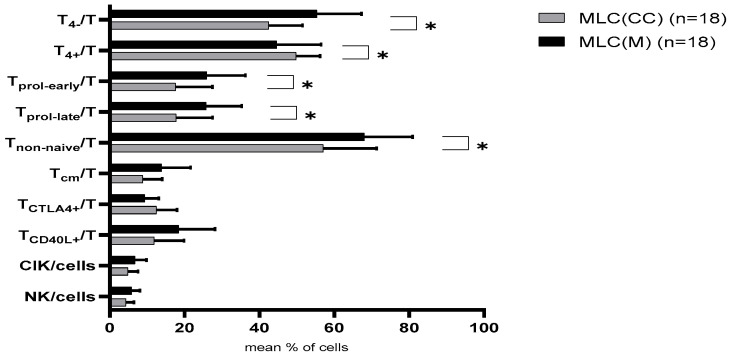
Effects of Kit-M-treated WB on the provision of immunoreactive cells after T-cell-enriched MLC. An aliquot of DC/DC_leu_ generated with vs. without Kit-M was cocultured with thawed autologous T-cells, stimulated with IL-2 and cultured for 6–7 days. Given are the mean frequencies ± standard deviation of T-cell subsets and of cells of the innate immune system after MLC(M) vs. MLC(CC), quantified by flow cytometry. Paired *t*-test was applied: results were considered as significantly different (*) with a *p*-value < 0.05. Abbreviations: T_4−_: CD8+ T-cells; T_4+_: CD4+ T-cells; T_prol-early_: proliferating T-cells-early; T_prol-late_: proliferating T-cells-late; T_non-naive_: non-naive T-cells; T_cm_: central (memory) T-cells; T_CTLA4_: CD152+ coexpressing T-cells; T_CD40L_: CD154+ coexpressing T-cells; CIK: cytokine-induced killer cells; NK: natural killer cells; *n*: number of cases. Cell subtypes are given in Supplementary Table S2.

After MLC, we found significantly higher frequencies of proliferating T-cells comparing MLC(M) with MLC(CC) (%T_prol-early_: Kit-M: 25.97 ± 10.28 vs. control 17.60 ± 9.80, *p* < 0.05; %T_prol-late_: MLC(M): 25.78 ± 9.47 vs. control 17.79 ± 9.66; *p* < 0.05). We also detected significantly higher frequencies of non-naive T-cells (%T_non-naive_/T: Kit-M: 68.03 ± 12.89 vs. control 57.07 ± 14.21, *p* < 0.05) in MLC(M) compared to MLC(CC). Furthermore, we detected significantly higher frequencies of CD8+ T-cells (%T_4−_/T: Kit-M: 55.39 ± 11.86 vs. control 42.45 ± 9.03, *p* < 0.05) and significantly lower frequencies of CD4+ T-cells (%T_4+_/T: Kit-M: 44.61 ± 11.85 vs. control 49.84 ± 6.36, *p* < 0.05) in MLC(M) compared to MLC(CC). No other significant results were obtained from the groups compared. However, a notable decrease in CTLA4-expressing T-cells and an increase in CD40L+ T-cells in Kit-M pretreated vs. untreated samples was found (%T_CD40L+_/T: Kit-M: 18.42 ± 9.71 vs. control 12.39 ± 7.39, *p* < 0.1) (Figure 3). We found comparable results in healthy samples (*n* = 5) (Supplementary Figure S1).

#### 3.2.2. (Significant) Activation of Immunoreactive (Leukemia-Specific) Cells After MLC (Compared to Before) in Patients’ and Healthy Donors’ Samples

An overall increase in immune cell activation was observed in whole blood (WB) samples following mixed lymphocyte culture (WBDC-MLC vs. WBDC and WBMDC-MLC vs. WBMDC), likely attributable to the stimulatory effect of IL-2. We detected Higher frequencies of proliferating T-cells (T_prol-early_/T), T_non-naive_/T, T_non-naive4+_/T_4+_, T_non-naive_/T, T_cm_/T and T_4−_/T were found within patients’ samples after comparison to before MLC (MLC(UC)) (Supplementary Figure S2). Within healthy donors’ and patients’ samples, we found higher frequencies of proliferating T-cells (T_prol-late_/T) and T_CD40L+_/T after MLC compared to before.

#### 3.2.3. Stimulatory Impact of Kit-M Treated WB on the Provision of Leukemia-Specific (Intracellular IFNγ and TNFα Producing or Degranulating) Immune Cells After MLC Detected by ICA and Deg Assays

We quantified intracellular IFN-γ and TNFα producing immunoreactive cells after MLC using Kit-M stimulated vs. not stimulated WB [(MLC(M)) vs. WB (MLC(CC)] using an intracellular cytokine assay (ICA) and a degranulation assay (Deg), as given in the Mat-Met section. The Deg assay detected the activity of degranulating immunoreactive cells, the ICA intracellularly IFNγ and TNFα producing immunoreactive cells after MLC(M) vs. MLC(CC)).

After MLC, cells were intracellularly stained with an antibody against IFNγ to identify cytokine-producing cells (according to the intracellular assay protocol. In analogy, degranulating immunoreactive cells were quantified after MLC(M) and MLC(CC) via the Deg assay, using an antibody against CD107.

**Figure 4:**
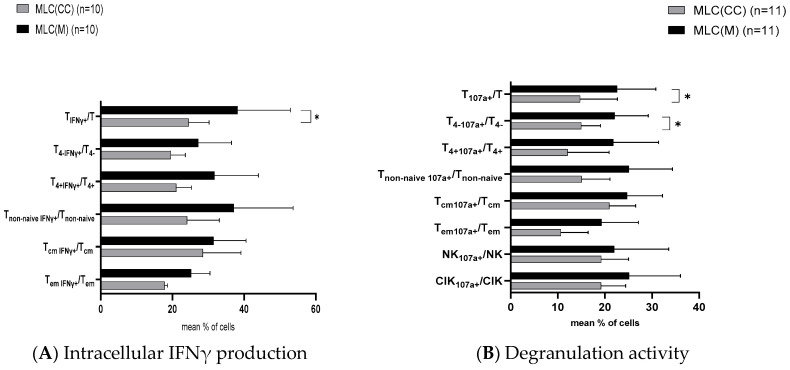
Effects of Kit-M-treated WB on the intracellular IFNγ production of T-cells (**A**) and on the degranulation activity of T-cells after MLC (**B**). Intracellular IFN-γ-producing or degranulating CD107a+ immune cells of Kit-M-pretreated WB (MLC(M)) and untreated WB (MLC(CC)) were evaluated using an ICA (**A**) or DEG assay (**B**) and quantified by flow cytometry. Given are the mean frequencies ± standard deviation of IFNγ producing (left side) or degranulating cell subsets (right side). Paired *t*-test was applied: results were considered as significantly different (*) with a *p*-value < 0.05. Abbreviations: T_cm_: central (memory) T-cells; Tem: effector (memory) T-cells; NK: natural killer cells; CK: cytokine-induced killer cells; *n*: number of cases. Cell subtypes are given in Supplementary Table S2.

We examined cells of the adaptive immune system and detected (significantly) higher frequencies of T_IFNγ+_/T, T_4+IFNγ+_/T_4+_ and T_4−IFNγ+_/T_4−_ in MLC(M) compared to MLC(CC) and uncultured WB (T_IFNγ+_/T: Kit-M: 38.16 ± 14.77% vs. control 24.46 ± 5.75%, *p* < 0.05; T_4+IFNγ+_/T_4+_: Kit-M: 31.70 ± 12.25% vs. control 21.03 ± 4.26%, *p* < 0.1; T_4−IFNγ+_/T_4−_: Kit-M: 27.20 ± 9.27% vs. control 19.53 ± 4.06%, *p* < 0.1) (Figure 4A). Additionally, we found significantly higher frequencies of T_non-naive IFNγ+_/T_non-naive_ and T_em IFNγ+_/T_em_ in cultured MLC (MLC(M) and MLC(CC)) compared to uncultured WB.

We detected (significantly) higher frequencies of T_TNFα+_/T, T_4+TNFα+_/T_4+_ and T_4−TNFα+_/T_4−_ in MLC(M) compared to MLC(CC) and WB (T_4+TNFα+_/T_4+_:Kit-M: 27.55 ± 10.79% vs. control 18.98 ± 5.13%, *p* < 0.1; and T_4−TNFα+_/T_4−_: Kit-M: 27.02 ± 9.14% vs. control 17.72 ± 7.66%, *p* < 0.05).

##### Effects of Kit-M-Treated WB on the Degranulation Activity of Immunoreactive Cells After MLC

In WB, (significantly) higher frequencies of T_107a+_/T, T_4+107a+_/T_4+_, T_4−107a+_/T_4−_, T_non-naive 107a+_/T_non-naive_, T_em+107a+_/T_em_ and CIK_107a+_/CIK were found comparing MLC(M) and MLC(CC) (T_4−107a+_/T_4−_:Kit-M: 22.07 ± 7.13% vs. control 14.96 ± 4.11, *p* < 0.05) (Figure 4B).

### 3.3. Improved Cytotoxicity Against Autologous Blasts of Kit-M Pretreated WB After MLC

After MLC, we used a CTX fluorolysis assay, as given in the Mat-Met section, to detect the stimulatory effect of Kit M-pretreated (vs. untreated) WB on the blast lytic activity after 3 h and 24 h of co-culture of immunoreactive cells (‘effector cells’) and blasts (‘target cells’). Achieved blast lytic activity was defined as the percentage difference in viable target cells (blasts) between the effector–target cell culture and the control [40]. ‘Improved lysis’ was calculated as the percentage difference between the achieved lysis of standard DC/DC_leu_ Kit-M pretreated vs. untreated WB in every given case [35].

**Figure 5:**
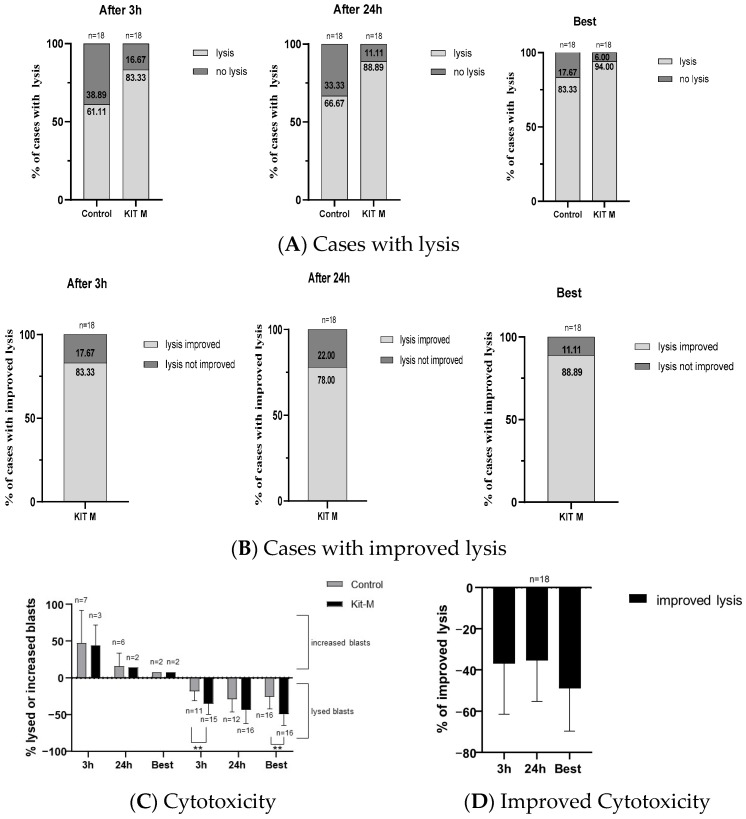
Effects of Kit-M-treated WB on the Induced Improved Cytotoxicity Against Autologous Blasts after MLC. Effects of Kit-M-treated WB on the improved cytotoxicity against autologous blasts after MLC, detected via cytotoxicity assay. After MLC of Kit-M treated vs. untreated WB, these (‘immune effector’) cells were mixed with blast-containing mononuclear cells (MNCs) (‘target cells’) and cultured for 3 h and 24 h. Given are the results after 3 h, 24 h, and the best achieved lysis after either 3 h or 24 h of the incubation time of the effector with target cells. The percentage of cases with lysis (**A**) and with improved lysis (**B**) compared to the control group (with target and effector cells mixed shortly before the measurement (Control)) is given. Given are the average ± standard deviation of achieved cytotoxicity (**C**) and improved cytotoxicity (**D**). Statistical significance was tested by a multiple-paired *t*-test. Results were considered as highly significant (**) with a *p*-value < 0.005. *n*: number of cases.

Using a CTX fluorolysis assay, Kit-M pretreatment resulted in improved cytotoxicity against autologous blasts, with blast lysis observed in a higher proportion of cases compared to controls after both 3 h (83.33 vs. 61.11%) and 24 h (88.89 vs. 66.67%) of coincubation (Figure 5A). Moreover, Kit-M–treated samples showed improved lysis in most cases compared to control (83.33% at 3 h; 78.00% at 24 h) (Figure 5B), with significantly higher lysis rates of blasts (34.43 vs. 17.88% at 3 h, *p* < 0.005). Furthermore, when comparing the best (after 3 OR 24 h of coincubation) achieved lysis rates, the Kit-M pretreated group exhibited 49.06% lysis compared to 25.42% in the non-pretreated group (*p* < 0.005) (Figure 5C).

In summary, we found more cases with achieved blast lysis as well as with improved lysis in Kit-M pretreated WB after MLC after 3 or 24 h of incubation time compared to the untreated control. Furthermore, we observed (significantly) higher frequencies of lysed blasts in MLC(M) vs. control after 3 and 24 h of incubation.

### 3.4. Correlation of Frequencies of DC_tol_ with Patients’ Response to Induction Chemotherapy Allocation to Risk Groups and Achieved Improved Cytotoxicity Against Autologous Blasts

We compared frequencies of DC_tol_ in Kit-M treated (vs. untreated) WB from AML patients with their response to induction chemotherapy and their allocation to ELN risk groups. In patients with low (*n* = 9) vs. high risk (*n* = 5), we observed non-significantly lower frequencies of DC_tol_. Moreover, we found lower frequencies of DC_tol_ in responders (*n* = 9) vs. non-responders (*n* = 9) to induction chemotherapy (Supplementary Figure S3).

**Figure 6:**
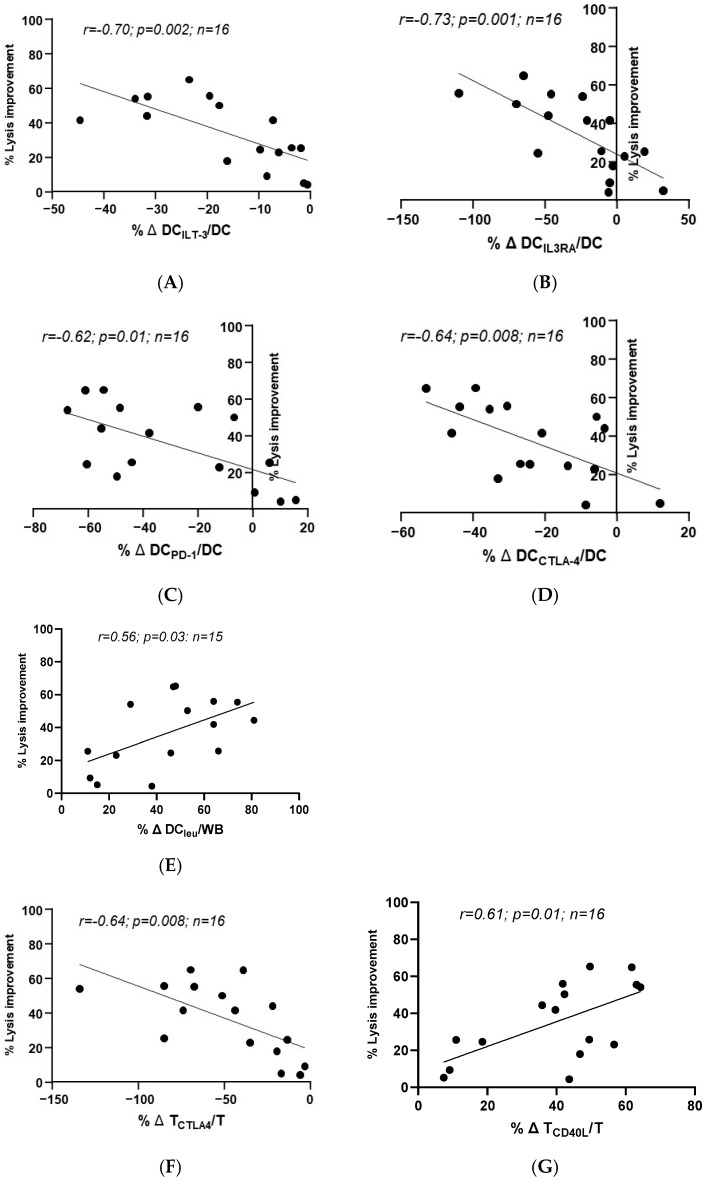
Correlation analyses of Kit-M stimulated (vs. unstimulated) DC or MLCs with later on achieved improvement of blast lysis. Correlations between lysis improvement and frequencies of DC subtypes (quantified by flow cytometry), relative to control, are shown for DC_ILT-3_ (**A**), DC_IL3RA_ (**B**), DC_PD-1_ (**C**), DC_CTLA4_ (**D**), DC_leu_ (**E**) after culture of WB with vs. without Kit-M. Corresponding correlations between lysis improvement for T-cell subtypes are shown for T_CTLA4_ (**F**) and T_CD40L_ (**G**) of Kit-M pretreated vs. untreated WB after MLC using MLC(M) and MLC(CC) as effector cells.

We correlated frequencies of DC_tol_ in Kit-M treated (vs. untreated) WB with improved cytotoxicity against autologous blasts after MLC. We found a significant negative correlation between DC_tol_ and achieved improved blast lysis in MLC(M) (compared to untreated) samples (e.g., Δ DC_ILT-3_/DC: r = −0.70; *p* = 0.002) (Figure 6A–D). We found a positive correlation between frequencies of DC_leu_ and improved blast lysis in MLC(M) (% Δ DC_leu_/WB: r = 0.56; *p* = 0.03) (Figure 6E). We found clear, although non-significant negative correlation between the frequencies of CTLA4-expressing CD3+ T-cells and improved blast lysis in MLC(M) (% Δ T_CTLA4+_/T: r = −0.64, *p* = 0.008) (Figure 6F), whereas we found a positive correlation between increased frequencies of T_CD40L+_/T and lysis improvement (r = 0.61; *p* = 0.01) (Figure 6G) with the blastolytic functionality and frequencies of CD40L+-expressing T-cells.

Blast lysis was quantified with a CTX fluorolysis assay using Kit-M treated or not pretreated MLC (MLC(M) and MLC(CC)) after co-culture with autologous blast containing MNC for 3 and 24 h. Lysis improvement was defined as the relative increase in lysed blasts in Kit-M–treated versus untreated control samples. Differences in cell subset frequencies are expressed as relative changes (Δ%) between Kit-M–treated and untreated conditions.

Given are the (*n*) number of cases, evaluated by Pearson correlation analyses. Correlation coefficients (r) and *p*-values (one-tailed) are given. Significance is defined as highly significant in cases with *p*-values < 0.005 and as significant with *p*-values < 0.05. Abbreviations for subtypes are given in Supplementary Table S2.

## 4. Discussion

### 4.1. DC-Based Immunotherapy in AML

Despite a significantly changed landscape of AML treatment (antibodies, DC-based immunotherapies, allogeneic stem cell transplantation or new chemotherapies (hypomethylating agents)), there is still a huge unmet need for sustaining remissions and preventing relapses [27,44]. Standard DC-based strategies use DC/DC_leu_ production with or without loading with leukemia-associated antigens (LAA) under good manufacturing practice (GMP), followed by adoptive transfer to patients [18,27]. Alternatively, (residual) blasts can be directly converted to DC_leu_ ex vivo using response modifiers like Kit-M (GM-CSF + PGE1) that activate (leukemia-specific/antileukemic) immune cells, which lead to improved cytotoxicity against autologous blasts—without induction of blasts’ proliferation [43,45]. Aslan-Rejeski et al. (2024) [10] have demonstrated the best achieved immunomodulatory effect of Kit-M (with respect to increase of (mature) DC_leu,_ and of activated and T effector and memory cells) using the established standard concentrations of PGE1 (1 µg/mL) and GM-CSF (800 U/mL GM-CSF), whereas lower and higher concentrations of PGE1 resulted in lower DC/DC_leu_ and improved cytotoxicity against autologous blasts. The immunomodulatory effects were observed exclusively when both response modifiers were applied in combination [10]. Moreover, studies by Atzler et al. (2024) and Filippini Velázquez et al. (2025) in selected therapy-refractory patients treated with Kit-M suggest that the induction of mature DC/DC_leu_ and subsequent leukemia-specific immune activation can also be achieved in vivo, going along with at least disease stabilization for some time, pointing to potential DC-mediated stabilization of the disease [46,47].

For a better understanding of DCs’ functionality and the potential of the DC-inducing drug Kit-M, it is crucial to examine the tolerogenic potential of DCs. In this ex vivo study, we therefore focused on the influence of Kit-M not only on the recruitment of immunogenic cells, but also on DC_tol_. Comparisons in this study between Kit-M-treated vs. untreated samples were performed using two-tailed paired student’s *t*-tests on dependent samples. Given the exploratory nature of the analyses, no correction for multiple testing was applied, acknowledging the increased risk of type I error.

### 4.2. Influence of Kit-M on the Generation of (Mature) DC/DC_leu_ and on Frequencies of DC_tol_

We confirm previous findings of our group, that treatment of (leukemic) WB with Kit-M produces mature DC or DC_leu_ without induction of proliferation of monocytes or blasts in healthy/leukemic samples [27,32,39,40,41] (Figure 1).

#### Decrease in DC_tol_ in Kit-M Treated WB

DC_tol_ plays an important role in regulating immune responses in healthy and diseased organisms [12]. Ex vivo-produced tolerogenic DC (DC_tol_) is used in the treatment of rheumatoid arthritis [48]. On the other hand, cancer cells benefit from (cancer-induced) tolerogenic properties of DC and the established tolerant microenvironment by exploiting these immunosuppressive mechanisms [49]. DC_tol_ can be characterized by the expression of different biomarkers. In this study, we focused on immunoglobulin-like transcript (ILT-) 3, cytotoxic T lymphocyte antigen-4 (CTLA4), programmed death one (PD-1) and interleukin-3 receptor (IL3RA) [9,17,20,24,50,51]. Since DC_tol_ are known to accumulate in AML patients, we tested whether Kit-M might reduce frequencies of ILT-3, CTLA4, PD-1 or IL3RA expressing DC_tol_ [52,53].

Since ILT-3 was found to be upregulated on DCs of cancer patients (whereas decreased in autoimmune disease) and is linked to immunosuppressive properties (e.g., inducing T-cell anergy), it was an interesting finding that Kit-M significantly decreased frequencies of DC_tol_ expressing ILT-3 [19,54,55,56] (Figure 2A). The ILT-3 signal transduction takes place via its immunoreceptor tyrosine-based inhibitory motifs (ITIMs), which suppress costimulatory stimuli. Through interaction with T-cells, ILT-3 induces regulatory T-cells (T_reg_), and thereby leads to an inert immune response [54]. Moreover, ILT-3-depleted DCs were shown to have opposite (immunogenic) functions [53]. DC_tol_ and regulatory T-cells work synergistically: suppressive T-cells induce the upregulation of ILT-3 on DCs, which in turn leads to T-cell anergy and causes the differentiation of regulatory/suppressive T-cells [19,56]. We have already shown that T_regs_ are (significantly) decreased after Kit-M pretreated vs. untreated MLC [34]. This mutual dependency could explain our finding that Kit-M leads to significantly decreased frequencies of DC_tol_ expressing ILT-3.

CTLA4 (CD152) is a well-known inhibitory costimulatory molecule. Its suppressive function mediated by T-cells has been extensively studied, while the role of CTLA4 expressed on DCs is not clear. Previous studies pointed out the tolerogenic effect of CTLA4 on DCs by downregulating the DC-maturation and antigen presentation [21]. The exact signaling pathway for their inhibitory role remains unclear. We were able to show that Kit-M leads to a decrease in CTLA4-expressing DC_tol_ compared to control (Figure 2A). As described previously, Kit-M induces DC-maturation. This effect could be attributed to significantly decreased frequencies of CTLA4-expressing DC_tol_ in Kit-M treated WB (compared to control).

Plasmacytoid dendritic cells (pDCs), a significant subtype of dendritic cells, strongly express CD123 (IL3RA) and are the main producers of IFNα. Therefore, they are a crucial link between the innate and adaptive immune systems. However, within the tumor microenvironment, pDCs might lose their immune-activating properties and can promote tumor progression [57,58]. Previous studies have demonstrated that CD123 is overexpressed in hematological malignancies such as AML [23]. In AML, patients’ pDCs were shown to secrete significantly less IFNα, concluding they might provide immune escape mechanisms in the tumor microenvironment through interactions with regulatory T-cells [57,58]. In addition, within the tumor microenvironment, tumor cells are capable of favoring DCs with immune-inert, regulatory properties [59]. In this study, we observed significantly less IL3RA-expressing DCs after Kit-M treatment (compared to control) (Figure 2A). Since it has been shown that the tolerogenic phenotype of pDCs can be reversed [14], we suggest that Kit-M can activate pDCs and therefore revert their tolerogenic function. Since pDCs promote the induction of T_regs_, our finding could explain previously published data that Kit-M leads to significantly fewer T_regs_ compared to control after MLC [34].

We found a significant decrease in DC_tol_ expressing ILT-3 and IL3RA (both markers are known to be upregulated in AML patients) [23,54] (Figure 2A). Non-significant effects of Kit-M on the provision of DC_tol_ were found in healthy samples (Figure 2B). We assume that Kit-M is able to revert tolerogenic effects in WB’s (tumor) microenvironment.

### 4.3. Provision of Immunoreactive Cells After Kit-M Treated WB

We observed (significantly) higher frequencies of activated immune cells after T-cell-enriched mixed lymphocyte culture (MLC) pretreated vs. not pretreated with Kit-M in patients’ and healthy samples (Figure 3), as shown before [34,43,45,60]. As published before, Kit-M treatment leads to (significantly) higher frequencies of T-cells and their subsets (e.g., T_prol-early_, T_prol-late_, T_non-naive_) after MLC compared to control [41,45,61] (Figure 3). The enhanced activation status of these T-cell subsets may help to overcome anergy of immunoreactive cells [62].

CTLA4 (CD152) on T-cells has been attributed to key functions in regulating immune responses: Among others, it raises the threshold for cytokine production and proliferation and inhibits cell cycle progression and transcription factors like NF-κB, NF-AT, and AP-1 [21]. Notably, we observed decreased frequencies of regulatory, CD152-expressing T-cells (T_CTLA4+_/T) in Kit-M treated vs. untreated WB (Figure 3). Since high frequencies of regulatory T-cells are associated with impaired anti-tumor immunity and disease progression [63], this finding suggests a shift towards a less immunosuppressive tumor environment. Furthermore, we were able to show higher frequencies of activated, CD154-expressing T-cells (T_CD40L+_/T) after Kit-M treatment compared to control (Figure 3), which are critical for B-cell maturation and support dendritic cell longevity via CD154-CD40 interactions [64,65,66], indicating enhanced anti-leukemic immune responsiveness.

Lymphocyte activation and DC maturation are further driven by the well-established cross-talk between DC and innate immune cells, including NK and CIK cells [67]. In line with this, we confirmed increased frequencies of NK and CIK cells after Kit-M treatment compared to controls (Figure 3), as previously reported [61], further supporting a less immunosuppressive milieu.

T-cells for MLC were enriched via CD3+-based positive cell selection, which may induce baseline activation. However, as both conditions (cultures with vs. without Kit-M) underwent identical isolation and culture procedures, any CD3+-related effects are expected to be comparable between groups. Thus, the observed differences are most likely attributable to Kit-M–mediated modulation of immune cell composition and function rather than the enrichment procedure itself.

While IFNγ producing and degranulating cells are regarded as ‘leukemia-specific cells’ (in case leukemic antigens are added to settings), our assay does not allow a strict distinction between the effects of adaptive and innate cells.

However, IFNγ producing and degranulating adaptive or innate cells can increase after stimulation with leukemic antigens—either added directly to cultures or provided by Kit-M–induced DC—as shown before [39]. These responses were observed in autologous systems (using patients’ blasts containing WB and T-cells). We did not use allogeneic blasts in our experiments, since we wanted to focus on leukemia-associated effects independent of HLA-mediated effects. In addition, previous (unpublished) data indicate that Kit-M does not induce autoreactive responses against monocytes, supporting its functional selectivity. Future studies incorporating specificity controls will be required to further dissect these mechanisms.

### 4.4. Increased IFNγ Production and Degranulation Activity in Cells After MLC in Kit-M Pretreated WB

ICA and Deg determine the (antigen-specific) activation status of immunoreactive cells by detecting cell degranulation and intracellular IFNγ [34]. As shown in Figure 4, we were able to observe increased frequencies of IFNγ producing non-naive and memory T-cells, which are linked to long-lasting antitumor immunity, after Kit-M treatment compared to control [44]. Since low doses of IFNγ are correlated with a higher risk of relapse in AML patients, the suppression of these immunosuppressive effects can be attributed to Kit-M, as higher frequencies of IFNγ-producing cells were found in Kit-M-treated vs. untreated WB [68].

As published before, we could detect increased frequencies of degranulating (CD107a+) cells (e.g., T_non-naive 107a+_), indicating the induction of leukemia-specific cells after Kit-M treatment [60] (Figure 4). Thus, we support previous studies stating improved cytotoxicity against autologous blasts after Kit-M treatment [69].

### 4.5. Improved Blastolytic Activity After MLC in Kit-M Treated WB

The most compelling evidence of induced or improved cytotoxicity against autologous blasts is the observation of increased blast lysis compared to controls (e.g., after MLC). This can be assessed through the chrome release, fluorolysis, or other assays [70]. It has already been demonstrated that Kit-M pretreatment of blast containing WB improves cytotoxicity of immunoreactive cells after MLC against autologous blasts, leading to significantly higher blast lysis compared to controls [34,40,43]. We observed increased numbers of cases exhibiting a superior blastolytic effect, as well as improved blast lysis from immune-reactive cells after MLC(M) compared to the control (Figure 5). In some cases, blast lysis was higher after 3 h of incubation of the target with effector cells, while in others, it was superior after 24 h. These variations may be due to different, independent blastolytic mechanisms: the faster perforin/granzyme pathways, which primarily result in blast lysis after 3 h of coincubation, and the slower Fas/FasL pathways, which are more effective after 24 h of coincubation [40,71]. Overall, our findings regarding the blastolytic effects support previous data, indicating that Kit-M pretreatment of WB enhances the blastolytic activity of immunoreactive cells ex vivo, although the selectivity of killing by innate or adaptive cells remains unknown [40,60].

### 4.6. Correlation of Improved Blastolytic Functionality with DCs and T-Cells

In terms of potential clinical applications for Kit-M, it is essential to assess whether pretreating whole blood with Kit-M enhances the cytotoxicity against autologous blasts mediated by specific cell subtypes. Comparison of lysis improvement (compared to control) and frequencies of DC_tol_ after Kit-M treatment showed a significant negative correlation between cases with achieved lysis and frequencies of DC_tol_ (Figure 6A–D)—and a positive correlation between lysis improvement and frequencies of DC/DC_leu_ (Figure 6E). This supports our hypothesis that we were able to reduce the frequencies of DC_tol_, correlating with improved cytotoxicity against autologous blasts in our settings, as published before [60].

Negative correlations were found for improved (Kit-M mediated) blast lysis and frequencies of regulatory T-cells (T_CTLA4+_/T), positive correlations with activated T-cells (e.g., T_CD40L+_/T) (Figure 6F,G), suggesting potential effects in improving blastolytic functionality via increased responsiveness of T-cells, leading to cytotoxicity against autologous blasts [66].

These findings also enhance our understanding of the role of different cellular subtypes in mediating (or suppressing) antileukemic responses, leading to improved cytotoxicity against autologous blasts [39,40], particularly in evaluating the immunological effects of novel therapies. Our results indicate that Kit-M treatment of leukemic whole blood, serving as an ex vivo model of in vivo simulation, downregulates immunosuppressive cells, enhances leukemia-specific immune activation, and improves blast lysis compared to controls. While these findings point towards potential in vivo effects of Kit-M (composed of approved drugs), direct clinical relevance remains to be established. Previous studies in therapy-refractory patients, either before [46] or after SCT [47] or of leukemically diseased rats, have shown leukemia-specific immune activation and memory responses and were associated with stabilization of the disease in patients or even significant blast reduction in leukemic rats. However, these observations remain preliminary due to the limited number of treated animals and human subjects.

### 4.7. Correlation of Results with Patients’ Allocation to Different Clinical Characteristics

We observed clear (although not significant due to low case numbers) correlations of lower frequencies of DC_tol_ and the patients’ allocation to the favorable (ELN) risk group, as well as a better response to induction chemotherapy (remission) (Supplementary Figure S3). Concerning DC/DC_leu_, we showed opposite effects.

DC_tol_ are characterized by modified cytokine production (low levels of secreting pro-inflammatory cytokines, high levels of secreting anti-inflammatory cytokines, e.g., IL-10, transforming growth factor β (TGF-β)) as well as displaying low levels of co-stimulatory and MHC molecules. These immunosuppressive mechanisms lead to T-cell anergy and the induction of T_reg_ differentiation [9,72]. Furthermore, DC_tol_ acquires the ability to induce T-cell death through the activity of the immunosuppressive enzyme indoleamine 2,3-dioxygenase (IDO), which is up-regulated in DC_tol_ [73,74].

DC vaccination in cancer immunotherapy is safe, can be combined with established therapies and provides an effective and promising tool to treat cancer [30]. As discussed in previous studies, DC vaccination might be limited by generating DCs that may induce immune tolerance via, e.g., inducing T_reg_ [27]. Kit-M has already been shown to reduce (leukemia-specific) regulatory effector cells after MLC ex vivo and even after treatment of refractory patients in vivo [34,47]. Here, we were able to successfully provide evidence that Kit-M pretreatment of blood leads to reduced frequencies of DC_tol_ and correlates with an improved cytotoxicity against autologous blasts. Thus, Kit-M (a combination of clinically approved drugs) might qualify for treatment of AML patients (with or without combination with established treatments) to increase and improve cytotoxicity against autologous blasts and to install leukemia-specific memory cells.

From a translational perspective, Kit-M may be particularly relevant in low disease burden settings, where residual leukemic blasts could be reprogrammed into functional DC/DC_leu_, thereby enhancing antileukemic immune responses/improve cytotoxicity against autologous blasts. While our ex vivo findings are encouraging, they require validation in prospective clinical studies to assess their clinical applicability in AML.

## 5. Conclusions

In conclusion, we demonstrated in this ex vivo study that we can down-regulate DC_tol_ while generating (mature) DC/DC_leu_ after treatment of leukemic whole blood with Kit-M, going along with the activation of immune cells after MLC—ultimately resulting in improved cytotoxicity against autologous blasts. Furthermore, we found a negative correlation between the Kit-M-mediated induced blast lytic effects and decreased DC_tol_, making Kit-M a promising tool for DC-vaccination, although our ex vivo system is limited due to missing organ interactions. Studies are currently being designed to investigate whether these cells could be effective in overcoming the inhibitory microenvironment in vivo.

## Data Availability

The data presented in this study are available in this article.

## Supplementary Materials

The following supporting information can be downloaded at: https://www.mdpi.com/article/10.3390/biomedicines14061279/s1. Table S1: Patients’ characteristics and healthy individuals. Table S2: Cell types evaluated by flow cytometry. Figure S1: Immunoreactive cell profiles from healthy donors after MLC. Figure S2: Immunoreactive cell profiles before and after MLC using leukemic WB. Figure S3: Correlation of DC_tol_ with patients’ allocation to ELN-risk groups (3A) and to response to induction chemotherapy (3B). Figure S4: Gating strategy of leukemia-derived dendritic cells (DC_leu_) via Flowcytometry. References in supplementary materials file can be found in [17,21,32,39,40,41,42,43,45,53,57,61,64,73,75,76].
