# Supplementary material for: Treatment of Leukemic Blood Samples with Granulocyte-Macrophage-Colony-Stimulating-Factor Combined with Prostaglandin E1 Is Associated with Reduced Frequencies of Tolerogenic Dendritic Cells and Increased Cytotoxicity Against Autologous Blasts
Source: Biomedicines. 2026 Jun 4;14(6):1279. doi: 10.3390/biomedicines14061279 (PMC13297566; doi:10.3390/biomedicines14061279)
Supplement: Supplementary file 1 [file biomedicines-14-01279-s001.zip › biomedicines-4228306-supplementary.pdf]

Supplementary Materials for

Treatment of Leukemic Blood Samples with Granulocyte-Macrophage-Colony-Stimulating-Factor Combined with Prostaglandin E1 Is Associated with Reduced Frequencies of Tolerogenic Dendritic Cells and Increased Cytotoxicity Against Autologous Blasts

Anne Hartz [1,2,*], Lin Li [1,2], Hazal Aslan Rejeski [1,2], Elena Pepeldjiyska [1,2], Elias Rackl [1,2], Tobias Baudrexler [1,2], Peter Bojko [2,3], Jörg Schmohl [2,4], Andreas Rank [2,5], Christoph Schmid [2,5] and Helga Schmetzer [1,2,*]

[1] Working-Group: Immune-Modulation, Department for Hematopoietic Cell Transplantation, Medical Department 3, Klinikum Grosshadern, Ludwig-Maximilians-University, 81377 Munich, Germany; lin.li0814@outlook.com (L.L.); hazlaslan@gmail.com (H.A.R.); elena.pepeldjiyska@gmail.com (E.P.); elias.rackl@hotmail.de (E.R.); tobsibaudrexler@googlemail.com (T.B.)

[2] Bavarian Cancer Research Center (BZKF), 91054 Erlangen, Germany; peter.bojko@swmbrk.de (P.B.); joerg.schmohl@diak-stuttgart.de (J.S.); andreas.rank@uk-augsburg.de (A.R.); christoph.schmid@uk-augsburg.de (C.S.)

[3] Department of Hematology and Oncology, Rotkreuzklinikum Munich, 80634 Munich, Germany

[4] Department of Hematology and Oncology, Diakonieklinikum Stuttgart, 70176 Stuttgart, Germany

[5] Department of Hematology and Oncology, University Hospital of Augsburg, 86156 Augsburg, Germany

* Correspondence: annsohart@gmail.com (A.H.); helga.schmetzer@med.uni-muenchen.de (H.S.)

Table S1. Patients' characteristics and healthy individuals.

diagn.	no.	age	sex	FAB type	stage	IC in WB (%)	blast phenotype [CD]	ELN (2017) risk stratification	Response to induction chemotherapy	experiments conducted
**AML**	1581	56	m	p/M4	dgn.	58	**34**, **117**, 33, 13, 15	low risk	yes	DCC; MLC; CTX; Deg/ICA
**AML**	1603	32	f	p/nd	dgn.	50	**34**, **117**, 15, 33	high risk	no	DCC; MLC; CTX; Deg
**AML**	1606	59	m	p/nd	dgn.	82	13, 33, **56,** 65**, 117**	low risk	yes	DCC; MLC; CTX; Deg
**AML**	1607	69	f	p/nd	dgn.	80	13, 33, 64, 65**, 117**	low risk	yes	DCC; MLC; CTX; Deg
**AML**	1608	61	f	p/nd	dgn.	23	13, 33, 34**, 117**, 56, **65**	low risk	yes	DCC; MLC; CTX; Deg
**AML**	1609	71	m	s/nd	dgn.	46	**117**, **56**, 65, 33, 13, 7	low risk	yes	DCC; MLC; CTX; Deg/ICA
**AML**	1610	82	f	s/nd	dgn.	14	**117**, 33, 14, **56**	low risk	yes	DCC; MLC; CTX; Deg/ICA
**AML**	1612	76	m	p/nd	dgn.	20	**34**, 65, 117, 133, 13	high risk	no	DCC; MLC; CTX; Deg/ICA
**AML**	1614	53	f	p/M4	dgn.	35	34, **117**, 14, 33, 13	interm. risk	yes	DCC; MLC; CTX; Deg/ICA
**AML**	1615	78	f	s/PMF	dgn.	53	**34**, **117**, 13	interm. risk	no	DCC; MLC; CTX
**AML**	1617	81	f	p/M1	dgn.	51	13, 33, 64, **34, 117**, 56	interm. risk	no	DCC; MLC; CTX; ICA
**AML**	1618	63	m	p/M1	dgn.	10	13, 15, **33**, **117**	low risk	yes	DCC; MLC; CTX; Deg/ICA
**AML**	1620	61	f	s/nd	dgn.	4	**34**, **117**, 33, 4	high risk	no	DCC; MLC; CTX
**AML**	1621	71	m	s/nd	dgn.	18	**34**, **117**, 5, 13	high risk	no	DCC; MLC; CTX; ICA
**AML**	1622	49	f	p/nd	dgn.	66	13, 33, **117**	low risk	yes	DC; MLC; CTX; ICA
**AML**	1627	58	f	p/nd	dgn.	36	33, **117**	interm. risk	no	DCC; MLC; CTX
**AML**	1584	80	m	p/nd	rel.	8	**34**, **117**, 65, 13	low risk	no	DCC; MLC; CTX; Deg/ICA
**AML**	1628	22	f	tAML/M5	rel. a. SCT.	14	**34**, **117**, 65, 33, 56, 64	high risk	no	DCC; MLC; CTX
**Healthy**	1582	27	f							DCC; MLC
**Healthy**	1583	28	m							DCC; MLC
**Healthy**	1611	26	f							DCC; MLC
**Healthy**	1613	24	f							DCC; MLC
**Healthy**	1619	25	m							DCC; MLC

Legend: dgn: diagnosis, AML: acute myeloid leukemia, no: patient's number, f: female; m: male; FAB type: French-American-British classification; M1: acute myeloblastic leukemia with minimal maturation, M4: acute myelomonocytic leukemia; M5: acute monocytic leukemia, p: primary AML, s: secondary AML; t: therapy induced = secondary, PMF: primary myelofibrosis; stage: dgn: first diagnosis, rel.: relapse, rel. a. SCT.: relapse after stem cell therapy, IC: immunocytologically determined, blast phenotype [CD]: CD, cluster of differentiation; the blasts markers used for expression analysis in each individual patient are highlighted in bold, ELN Risk Stratification at initial diagnosis, DCC: dendritic cell culture; MLC: mixed lymphocyte culture measurement; Deg: Degranulation Assay; ICA: Intracellular assay; CTX: cytotoxicity (fluorolysis) assay; nd: no data.

Table S2. Cell types evaluated by flow cytometry.

Cell Type	Name of Subgroups	Abbreviation of Subgroups	Surface Marker	Referred to	Abbreviation	Reference
**Subtypes of blasts and DC**						
**Blasts**	Leukemic blasts	BLA	BLA (e.g. CD34+, CD117+)	WB	BLA/WB	[42]
	Proliferating blasts	$BLA_{prol-CD71}$	BLA+DC-CD71+	BLA	$BLA_{prol-CD71}$/BLA	[43]
	Proliferating blasts	$BLA_{prol-IPO38}$	BLA+DC-IPO38+	BLA	$BLA_{prol-IPO38}$/BLA	[43]
**Dendritic cells**	Dendritic Cells	DC	DC+ (CD80+, CD206+)	WB	DC/WB	[41]
	Leukemia derived DC	DC_{leu}	DC+BLA+	WB<br>DC<br>BLA	DC_{leu}/WB<br>DC_{leu}/DC<br>DC_{leu}/BLA	[41]
	Mature DC	DC_{mat}	DC+CD197+	WB<br>DC	DC_{mat}/WB<br>DC_{mat}/DC	[41]
	Mature DC_{leu}	$DC_{leu-mat}$	DC+BLA+CD197+	WB<br>DC<br>DC_{leu}<br>DC_{mat}<br>BLA	$DC_{leu-mat}$/WB<br>$DC_{leu-mat}$/DC<br>$DC_{leu-mat}$/DC_{leu}<br>$DC_{leu-mat}$ /DC_{mat}<br>$DC_{leu-mat}$/BLA	[41]
	Tolerogenic DC	DC_{tol}<br>DC_{ILT-3}<br>DC_{IL3RA}<br>DC_{CTLA4}<br>DC_{PD-1}	(e.g. CD85k/CD123/CD152/CD279)<br>CD85k+DC+<br>CD123+DC+<br>CD152+DC+<br>CD279+DC+	DC<br>DC<br>DC<br>DC<br>DC	DC_{tol}/DC<br>DC_{ILT-3}/DC<br>DC_{IL3RA}/DC<br>DC_{CTLA4}/DC<br>DC_{PD-1}/DC	[73]<br>[53]<br>[57]<br>[21]<br>[17]
**Subtypes of Immune Reactive Cells**						
**T-cells**	CD3+ pan T-cells	T	CD3+	cells	T/cells	[45]
	CD4+ coexpressing T-cells	T_{4+}	CD3+CD4+	T	T_{4+}/ T	[45]
	CD8+ coexpressing T-cells	T_{4-}	CD3+CD8+	T	T_{4-}/ T	[45]
	Proliferating T-cells-late	$T_{prol-late}$	CD3+CD71+	T	$T_{prol-late}$/T	[45]
	Proliferating T-cells-early	$T_{prol-early}$	CD3+CD69+	T	$T_{prol-early}$/T	[45]
	Non- naïve T-cells	$T_{non-\ naïve}$	CD3+CD45RO+	T	$T_{non-\ naïve}$ /T	[40]
	Central (memory) T-cells	T_{cm}	CD3+CD45RO+CD197+	T	T_{cm}/T	[40]
	CD154+ coexpressing T-cells	T_{CD40L+}	CD3+CD154+	T	T_{CD40L}/T	[64]
	CD152+ coexpressing T-cells	T_{CTLA4+}	CD3+CD152+	T	T_{CTLA4}/T	[75]
**Cytokine induced killer cells**	CD3+CD56+ CIK cells	CIK	CD3+CD56+	cells	CIK/cells	[61]
**Natural killer cells**	CD3-CD56+ NK cells	NK	CD3-CD56+	cells	NK/cells	[61]

**Subtypes of different degranulating (CD107a+) cells**						
**T-cells**	CD3+ pan T-cells	T_{107a+}	CD107a+CD3+$_{+}$	T	T_{107a+}/T	[76]
	CD4+ coexpressing T-cells	$T_{4+107a+}$	CD107a+CD3+CD4+	T_{CD4+}	$T_{4+107a+}/T_{4+}$	[76]
	CD8+ coexpressing T-cells	$T_{4-107a+}$	CD107a+CD3+CD4-	T_{CD4-}	$T_{4-107a+}/T_{4-}$	[76]
	Non- naïve T-cells	$T_{non\text{-}\ naïve\ 107a+}$	CD107a+CD3+CD45RO+	$T_{non\text{-}naïve}$	$T_{non\text{-}naive107a+}/T_{non\text{-}\ naïve}$	[76]
	Effector (memory) T-cells	$T_{em107a+}$	CD107a+CD3+CD45RO+CD197-	T_{em}	$T_{em107a+}/T_{em}$	[76]
	Central (memory) T-cells	$T_{cm107a+}$	CD107a+CD3+CD45RO+CD197+	T_{cm}	$T_{cm107a+}/T_{cm}$	[76]
**Cytokine induced killer cells**	CD3+CD56+CIK cells	CIK_{107a+}	CD107a+CD3+CD56+	CIK	CIK_{107a+}/CIK	[76]
**Natural killer cells**	CD3-CD56+NK cells	NK_{107a+}	CD107a+CD3-CD56+	NK	NK_{107a+}/NK	[76]
**Subtypes of different intracellularly IFNγ or TNFα-producing cells**						
**T-cells**	CD3+ pan T-cells	$T_{IFN\gamma +/TNF\alpha}$	IFNγ+/TNFα+CD3+	T	$T_{IFN\gamma+/TNF\alpha}$/T	[40]
	CD4+ co-expressing T-cells	$T_{4+IFN\gamma +/TNF\alpha}$	IFNγ+/TNFα+CD3+CD4+	T_{CD4+}	$T_{4+IFN\gamma+/TNF\alpha}/T_{4+}$	[40]
	CD8+co-expressing T-cells	$T_{4\text{-}\ IFN\gamma+/TNF\alpha}$	IFNγ+/TNFα +CD3+CD4-	T_{CD4-}	$T_{4\text{-}IFN\gamma+/TNF\alpha}/T_{4-}$	[40]
	Non- naïve T-cells	$T_{non\text{-}\ naïve\ IFN\gamma+/TNF\alpha}$	IFNγ+/TNFα+ CD3+CD45RO+	$T_{non\text{-}naïve}$	$T_{non\text{-}naïveIFN\gamma+/TNF\alpha}$ $/T_{non\text{-}\ naïve}$	[40]
	Effector (memory) T-cells	$T_{em\ IFN\gamma+/TNF\alpha}$	IFNγ+/TNFα +CD3+CD45RO+CD197-	T_{em}	$T_{emIFN\gamma+/TNF\alpha}/T_{em}$	[40]
	Central (memory) T-cells	$T_{cm\ IFN\gamma+/TNF\alpha}$	IFNγ+/TNFα +CD3+CD45RO+CD197+	T_{cm}	$T_{cmIFN\gamma+/TNF\alpha}/T_{cm}$	[40]

Supplementary Figures

Figure S1. Immunoreactive cell profiles from healthy donors after MLC.

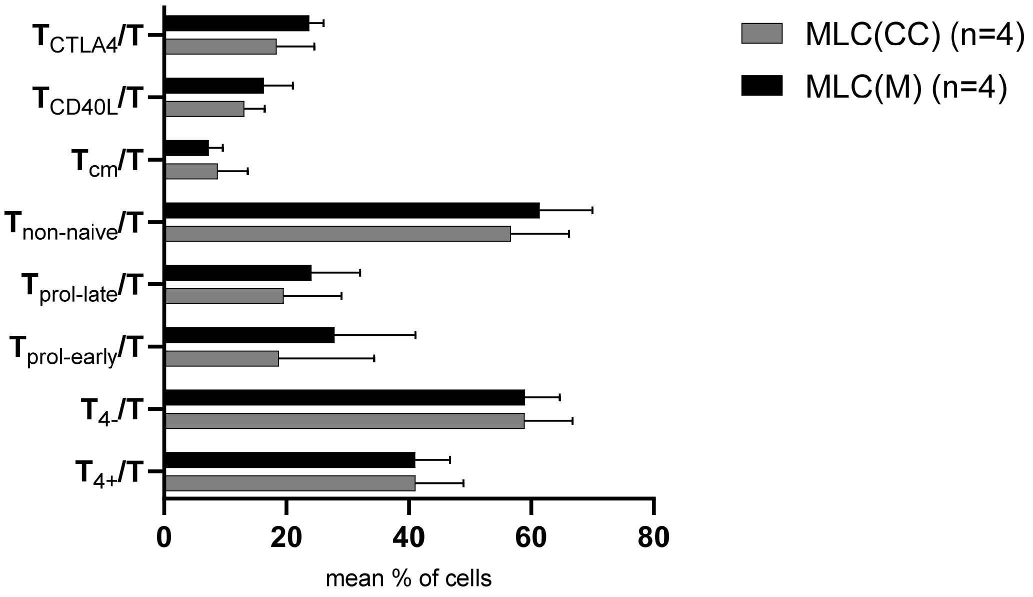

Figure S1: Mean frequencies ± standard deviation of T-cell subsets after stimulation of T-cell-enriched immunoreactive cells containing Kit-M-pretreated WB (MLC(M)) from healthy T-cells compared to WB not pretreated with Kit-M (MLC(CC)). Paired t-test was applied.

Abbreviations: T_{4-}: CD8+ T-cells; T_{4+}: CD4+ T-cells ; $T_{prol\text{-}early}$: proliferating T-cells-early; $T_{prol\text{-}late}$: proliferating T-cells-late; $T_{non\text{-}naïve}$: non-naïve T-cells; T_{cm}: central (memory) T-cells; T_{CTLA4}: CD152+ coexpressing T-cells; T_{CD40L}: CD154+ coexpressing T-cells; CIK: cytokine-induced killer cells; NK: natural killer cells; n: number of cases. Cell subtypes are given in Supplementary Table S2.

Figure S2: Immunoreactive cell profiles before and after MLC using leukemic WB

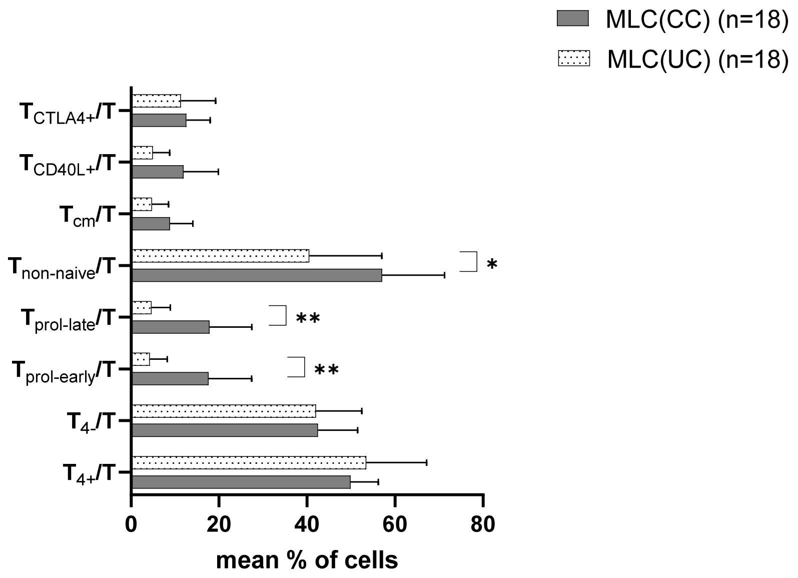

Figure S2: Composition of immunoreactive cells before and after T-cell-enriched MLC using leukemic WB without Kit pretreatment. Cells were analysed before and after 7 days of MLC using leukemic WB without Kit pretreatment and IL-2. Cells before MLC from leukemic WB without added Kit-M (MLC(UC)) and cells after MLC without added Kit-M (MLC(CC)) are given. Paired t-test was applied: results were considered as significantly different(*) with a p-value <0.05 and as highly significant (**) with a p-value <0.005.

Abbreviations: T_{4-}: CD8+ T-cells; T_{4+}: CD4+ T-cells ; $T_{prol\text{-}early}$: proliferating T-cells-early; $T_{prol\text{-}late}$: proliferating T-cells-late; $T_{non\text{-}naïve}$: non-naïve T-cells; T_{cm}: central (memory) T-cells; T_{CTLA4}: CD152+ coexpressing T-cells; T_{CD40L}: CD154+ coexpressing T-cells; CIK: cytokine-induced killer cells; NK: natural killer cells; n: number of cases. Cell subtypes are given in Supplementary Table S2.

Figure S3. Correlation of DC_{tol} with patients' allocation to ELN-risk groups (3A) and to response to induction chemotherapy (3B)

A: ELN-Risk after DC Culture **B: Responders after DC Culture**

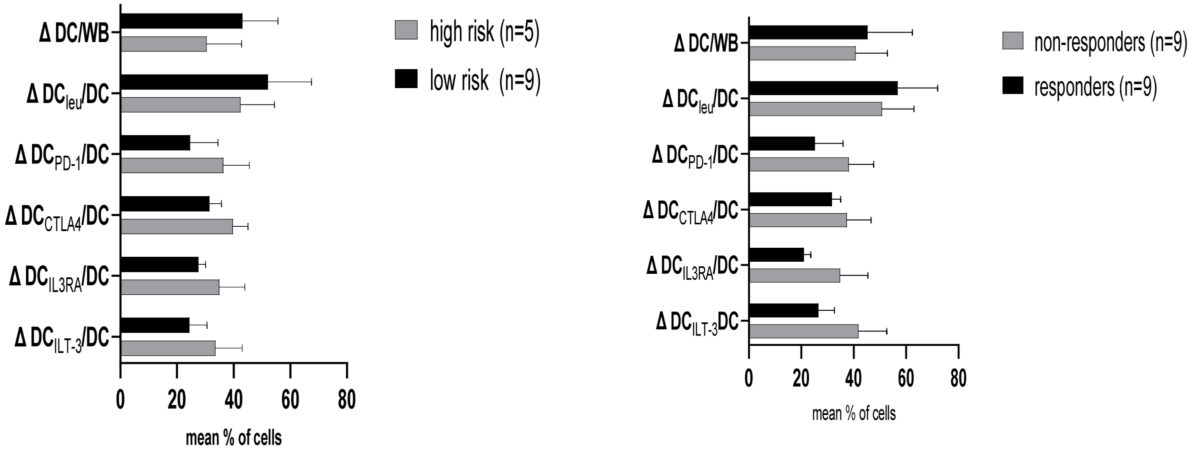

Figure S3: DC_{tol} and DC/DC_{leu} within patients with different clinical characteristics: (A) illustrates the average frequencies ± standard deviation of DC_{tol} and DC/DC_{leu} after DC cultivation with vs without Kit-M divided into two groups considering clinical ELN risk stratification (high vs low); (B) illustrates the average frequencies ± standard deviation of DC_{tol} and DC/DC_{leu} after DC cultivation with vs without Kit-M divided into two groups considering the response to induction chemotherapy. Differences in cell subset frequencies are expressed as relative changes (Δ%) between Kit-M–treated and untreated conditions. Abbreviations for cell subtypes are given in Supplementary Table S2.

Figure S4: Gating strategy of leukemia derived dendritic cells (DC_{leu}) via Flowcytometry:

1) Gating strategy for DC and DC_{leu} after 7d culture of a leukemic WB sample with vs without Kit-M

Frequencies of DC_{leu} in a leukemic WB sample without added Kit-M (Control)

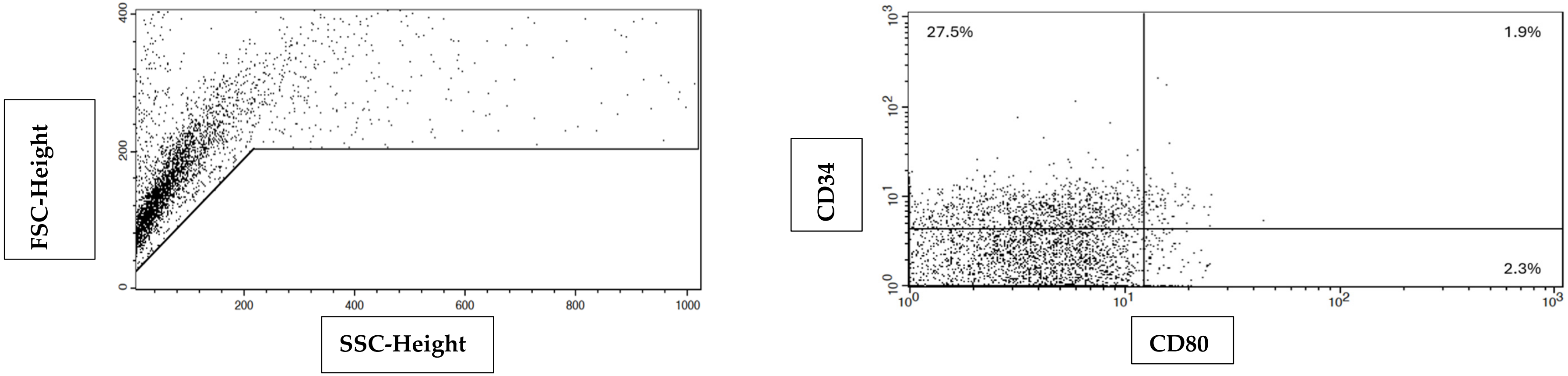

Frequencies of DC_{leu} in a leukemic WB sample after treatment with Kit-M

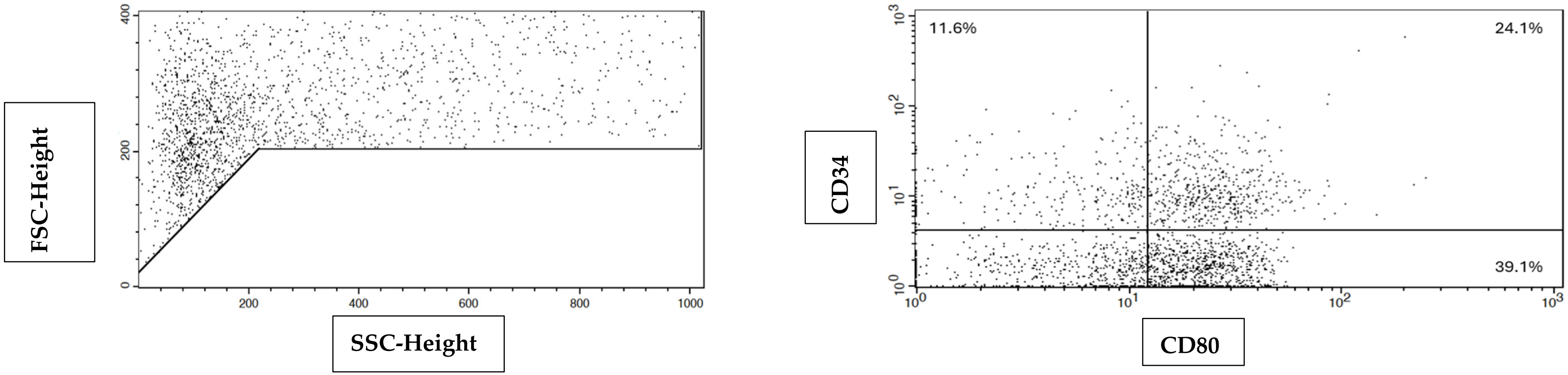

Gates were set around blasts and myeloid cells and debris excluded. Cells are presented in **forward scatter (FSC) and side scatter (SSC)** presentations (left side). In a next step frequencies of blasts (positive for CD34) and DC (positive for CD80) are given in a dot plot (right side). DC without coexpression of leukemic antigens and of DC_{leu}, expressing both antigens can clearly be differentiated.

All together a shift from blasts to leukemia derived DC under the influence of Kit-M is visible.

Materials and Methods

Flow Cytometry

Flow cytometric analyses were performed to characterize and quantify frequencies, phenotypes and subsets of leukemic blasts, DCs, monocytes, B-, T-, NK- and CIK cells before and after cultures. After staining, cells were examined using a FACSCalibur four channel cytometer. The respective antibodies were labeled with fluorescein-isothiocyanate (FITC), phycoerythrin (PE), phycoerythrin/ cyanine 7 (PE/Cy 7) and allophycocyanin (APC). The antibodies were provided by Beckman Coulter ([a], Brea, California, US), BD Biosciences ([b], San Jose, California, US), BioLegend ([c], San Diego, California, US) and Santa Cruz Biotechnology ([d], Dallas, Texas, US). To determine the amount of viable cells, 7-AAD Staining Solution (7AAD, BD Biosciences, San Jose, California, US) was used.

FITC-conjugated antibodies were used against: IgG[a], CD34[a], CD65[a], CD33[a], CD117[c], CD15[a], CD56[c], CD3[b], CD71[a], ipo38[d], CD19[a], CD107a[c], CD4[b], CD45RO[a], CD14[a]. The antibodies used for PE were against: IgG[a], CD117[a], CD80[a], CD56[a], CD206[a], CD279[b], CD152[b], CD123[c], CD3[a], IFNγ[c], CD4[b]. Antibodies against IgG[a], CD15, CD117[a], CD19[a], CD34[a], CD197[b], CD56[a], CD4[a], TNFα[c], CD3[a] and CD14[b] were labeled with PE/Cy 7. APC-connected antibodies were used against: IgG[a], CD206[b], CD80[c], CD34[a], CD117[a], CD14[a], CD56[a], CD69[b], CD45RO[c], CD4[b], CD3[a], CD19[a].

Before staining, erythrocytes in WB samples were lysed with lysing solution (BD Biosciences, San Jose, California, US) following manufacturer's instructions. After suspending the remaining cells in PBS/FBS to prevent unspecific binding, staining was carried out by adding the corresponding antibodies. After 15 minutes of incubation at room temperature in the dark, the cells were washed, centrifuged and resuspended in 100 µl PBS. To determine intracellular markers (e.g. ipo38, ICA), the FIX & PERM Cell Fixation and Cell Permeabilization Kit (ThermoFisher Scientific, Darmstadt, Germany) was used. Isotype samples were used as control [40].

Initial Sample Preparation

The WB samples were mixed with lysing solution (BD Biosciences, San Jose, California, US), washed and centrifuged. Afterwards the samples were resuspended in phosphate-buffered saline (PBS, Sigma-Aldrich, St. Louis, Missouri, US) consisting of 5% fetal bovine serum (FBS, Bio&Sell, Feucht, Germany). The acquired cells were stained with antibodies.

Furthermore, MNCs were separated from the WB by using a density gradient centrifugation using the Ficoll-Hypaque-Technique (density of separating solution 1.077 g/mL, Biocoll Separating Solution, Bio&Sell, Feucht, Germany, article number: BS.L.6715) and afterwards resuspended in RPMI-1640 (RPMI, PAN-Biotech, Aidenbach, Germany) consisting of 100 U/ml penicillin and 0.1 mg/ml streptomycin (PS, PAN-Biotech, Aidenbach, Germany). An aliquot part of the MNCs was frozen, the other part was used for separating CD3+ T-cells by applying the MACS-microbead-technology (Milteny Biotech, Bergisch Gladbach, Germany)[33]. The enriched T-cells were frozen for subsequent experiments.

The obtained cells were frozen in a medium containing 70% RPMI and MNCs, 20% human serum (human serum, PAN-Biotech, Aidenbach, Germany) and 10% dimethyl sulfoxide (DMSO, Sigma-Aldrich, St. Louis, Missouri, US). The suspension was mixed and stored at -80°C. For defrosting, the cells were placed in a 38°C water bath, suspended in RPMI and human serum, centrifuged and resuspended in the medium according to the subsequent experiments [40,39].

Dendritic Cell Culture

DC/DC_{leu} were generated from WB from AML patients in acute phases of the disease or healthy donors. In accordance to the protocols 500 µL of WB, diluted with 500 µL X-Vivo-15-medium (Lonza, Basel, Switzerland), were pipetted into 24-multiwell-tissue-culture-plates (Thermo Fisher Scientific, Darmstadt, Germany). For generating DC_{leu}, response modifiers (Kit-M) were added on day 0 and after 2-3 days after incubation under physiological conditions at 37°C, 5% CO_2, 21% O_2 and 95% humidity. For control, WB without Kit-M was cultivated. The cells were harvested after 6-8 days and used for following experiments[14]. Before and after cell culture DC-subsets were quantified by flow cytometry.

Kit-M:

Kit-M contains GM-CSF (800 U/ml, granulocyte macrophage colony stimulating factor, Sanofi-Aventis, Frankfurt, Germany) and PGE1 (1 µg/ml, prostaglandin E1, Santa Cruz Biotechnology, Dallas, Texas, US). Incubation and stimulation was handled as described above.

In the following, cells will be referenced as DC(C) for control with untreated WB and DC(M) for Kit-M-treated WB [40].

Mixed Lymphocyte Culture

The cultivation of MLC started after harvesting DC cultures after 7 days. For the cultivation of cells in MLCs, frozen T-cells from AML patients or healthy volunteers were defrosted and $1x10^6$ positively selected CD3+ T-cells were added per well into a 24-well-plate. The cells were co-cultured with a stimulator cell suspension consisting of $2.5x10^5$ DC/DC_{leu} generated with Kit-M (MLC(M)). DC/DC_{leu} without pretreatment with Kit-M served as control (MLC(C)). Afterwards, each well was filled up to a total volume of 1ml with RPMI/PS medium. Besides 50 U/ml IL-2 (PeproTech, Berlin, Germany) was added on the first day and after 2-3 days of culture. All cells were incubated under physiological conditions at 37°C, 21% O_2 and 5% CO_2. The MLCs were harvested after 6-7 days, cell subtypes were quantified and used for subsequent experiments such as degranulation assay (Deg), intracellular cytokine assay (ICA) and cytotoxicity fluorolysis assay (CTX) [40].

Degranulation Assay

This assay served to detect cell degranulation activity of different lymphocyte subsets by using a FITC-conjugated antibody against CD107a, a lysosomally associated membrane glycoprotein 1 (BioLegend, San Diego, California, US). The Deg was conducted with cells after MLC on day 14 from AML patients.

T-cell enriched cells, stimulated with or without Kit-treated WB after MLC (further referred to as MLC(M)), were put in a 96-well-plate (Becton Dickinson, Heidelberg, Germany). RPMI medium was added. After one hour of incubation under physiological conditions at 37°C, 5% CO_2, 21% O_2 and 95% humidity, Monensin Solution (final concentration: 2 µmol/l, BioLegend, San Diego, California, US) was mixed with the cultures to avoid the loss of FITC-CD107a antibodies' fluorescence after reinternalization in endosomal and lysosomal cell compartments. After another 15 hours of incubation, the cells were harvested and prepared for analysis by the flow cytometry [39].

Intracellular Cytokine Assay

This assay was performed to detect the intracellular production of interferon gamma (IFNγ) and tumor necrosis factor (TNFα) in WB and in MLC(M). Comparable to the Deg, cells mixed with RPMI medium were put in a 96-well-plate (Becton Dickinson, Heidelberg, Germany) and incubated for an hour under standard culture conditions at 37°C, 5% CO_2, 21% O_2 and 95% humidity. Afterwards Brefeldin A solution (final concentration: 5 µg/ml, BioLegend, San Diego, California, US) was added to the wells following manufactors' instructions to avoid premature cytokine secretion. After another 15 hours of incubation, cells were harvested and prepared for flow cytometric analyses [39].

Cytotoxicity Fluorolysis Assay

The CTX was conducted to analyze the lytic activity of MLC-stimulated T-cell enriched immunoreactive cells (with or without Kit-treated WB after MLC) against leukemic blasts (MNCs containing patients' blasts). Equal amounts of effector- and target-cells were used and a medium consisting of 85% RPMI/PS medium and 15% human serum was added. These co-cultured cells served as main sample and were then incubated for 0, 3 or 24 hours under physiological conditions at 37°C, 5% CO_2, 21% O_2 and 95% humidity. For the control sample, effector- and target-cells were put separately in the incubator and mixed shortly before measuring. To determine the cell viability, 7-AAD Staining Solution (7AAD, BD Biosciences, San Jose, California, US) was added after the corresponding incubation times. After putting an established amount of Flow-Count Fluorospheres (Beckman Coulter, Brea, California, US) into the tubes, flow cytometric analyses were performed to quantify the target-cells. 'Blast lysis' was described as the reduction of viable blasts compared in the main and the control sample. 'Lysis improvement' was defined by comparing 'blast lysis' with and without Kit-treated WB [40]. For a more detailed assessment, 'improved lysis' was calculated as the percentage difference between the achieved lysis of standard DC/DC_{leu} Kit-M pretreated WB vs. untreated WB in every given case, as described before [32,45].
